# Prevalence of impaired functional reading ability and its association with quality of life, daily activity, mobility and social participation among general older adults in Germany

**DOI:** 10.1186/s12877-019-1191-2

**Published:** 2019-06-25

**Authors:** Franziska Radicke, Thea Schwaneberg, Claudia Meinke-Franze, Clemens Jürgens, Hans Jörgen Grabe, Wolfgang Hoffmann, Frank Tost, Neeltje van den Berg

**Affiliations:** 1 Department Epidemiology of Health Care and Community Health, Institute for Community Medicine, University Medicine Greifswald, Ellernholzstraße 1-2, D-17487 Greifswald, Germany; 2 Department of Study of Health in Pomerania / Clinical-Epidemiological Research, Institute for Community Medicine, University Medicine Greifswald, Greifswald, Germany; 3 Department of Ophthalmology, University Medicine Greifswald, Greifswald, Germany; 4 Department of Psychiatry and Psychotherapy, University Medicine Greifswald, Greifswald, Germany; 5 Medical Center Hamburg-Eppendorf (UKE), Hamburg, Germany

**Keywords:** Functional reading ability, Elderly, Study of health in Pomerania, Nieden reading chart, Daily activities

## Abstract

**Background:**

The prevalence of visual impairment and of impaired functional reading ability rapidly increase with age. However, functional reading ability is essential for an autonomous lifestyle. We analyzed the prevalence of impaired functional reading ability in the general elderly population and the association of impaired functional reading ability with quality of life, daily activities, mobility, and social participation.

**Methods:**

Cross-sectional data from a population-based cohort were analyzed. Participants aged ≥65 years were tested for their functional reading ability using (1) Nieden charts (cognitive reading ability) and (2) a test in which a telephone number had to be found (reading comprehension). Prevalences of impaired functional reading ability were calculated. In multivariable regression models, the associations of cognitive reading ability (1) with quality of life, daily activities, mobility, and social participation were examined.

**Results:**

60 of 780 participants (7.69%) were able to read the third last text of the Nieden test (good cognitive reading ability), whereas 7 participants (0.9%) were unable to read any of the texts. 716 participants (91.8%) identified the phone book entry successfully (good reading comprehension). Multivariable regression models revealed no significant associations of the cognitive reading ability (1) with quality of life, daily activities, social participation, and mobility.

**Conclusion:**

Our results showed a high prevalence of impaired cognitive reading ability (1). Reading comprehension (2) was slightly affected. The loss of cognitive reading ability usually progresses over years; signs and symptoms might remain unrecognized when compensated by other functions.

## Background

A key determinant of a self-determined and autonomous lifestyle of elderly people is health. The World Health Organization (WHO) developed the International Classification of Functioning, Disability and Health (ICF) to comprehensively describe the functional health status of individuals [1]. The ICF consists of two parts: a. functioning and disability and b. contextual factors. Physical functions are a component of the first part. Vision is listed in the section of sensory abilities (of the physical functions) in the ICF.

The risk of impaired vision increases with age [2, 3]. In Germany, 37% of the women and 29% of men aged ≥65 years report vision problems or blindness (assessed via CATI: computer assisted telephone interviewing). Participants were asked if they: a) can read a printed newspaper, and b) can recognize the face of a person 4 m away, e.g. across the street (if necessary with the help of a viewing aid). [4]. Study data from other countries are partially similar, e.g. for Poland (30.8% of the persons ≥60 years reported visual impairment) [2]. For other countries, a lower prevalence is shown. In the USA 14.4% of the ≥60 years were visually impaired [5]. A British investigation yielded prevalences of visual impairment between 12.4 and 19.9% of those aged ≥75 years depending on the definition adopted [6]. The prevalence in women was higher than in men of similar ages in all mentioned studies.

Impaired vision can affect health, daily activities, and social participation [7, 8]. Visual impairment is associated with a higher number of falls [9, 10], limited mobility [11], worse balance, fall-related hip fractures [12, 13], a poorer health-related quality of life [14] and a higher rate of physical and mental comorbidities [15]. These associations work both ways - and the direction of causality is not easy to assess.

The ability to read is an important determinant for an independent and autonomous life style, not only for the elderly. This includes not only the visual acuity, but also the functional ability to understand and interpret the content of written text. Impaired functional reading ability (measured by a functional vision questionnaire) influences balance and mobility [16] and often leads to restrictions in daily activities and social participation.

In this analysis, we examined the prevalence of impaired functional reading ability in general older adults in the northeast of Germany and its association with daily activities, quality of life, mobility, falls, and social participation.

## Methods

### Data and measurements

The data for this analysis were retrieved from the Study of Health in Pomerania (SHIP). SHIP is a population-based cohort study conducted in the region Western Pomerania in the northeast of Germany. Goal of the SHIP-study is to find prevalences of diseases, functional limitations and risk factors as well as associations and causalities between risk factors and diseases and functional limitations. At baseline (1997–2001), a representative sample of 4308 participants was examined. The baseline assessment contended of a range of interviews, self-reported questionnaires, and physical examinations. All participants aged ≥65 years (*n* = 780, mean age 72.8 ± 5.9 years) underwent a standardized investigation of their functional reading ability. Other relevant factors for this analysis were obtained from self-reported questionnaires (quality of life, falls, mobility, daily activities) and structured, computer-based interviews (social participation, cognition) using standardized questionnaires. After the baseline assessment, 5 and 10 year follow-up examinations were conducted. All participants gave informed consent to the study and scientific use of the data. The study was approved by the ethics committee of the University Medicine Greifswald. More details on the design and contents of the SHIP cohort study are published elsewhere [17–22]. The data for this analysis were retrieved from the 10-year follow-up of the SHIP cohort study.

#### Functional reading ability

The assessment of the functional reading ability included two tests: (1) Nieden’s reading charts [23] for the assessment of cognitive reading ability (composed of reading acuity and reading speed), and (2) finding and reporting a pre- determined entry on a phone book page to assess the reading comprehension. The Nieden reading charts are a well-known instrument in everyday clinical practice. They are not often used for scientific analyses. However, they offer the possibility to measure the cognitive reading ability.

(1) Nieden’s reading chart was placed at a reading distance of 40 cm (15.75 in.) [24] on a bookend on a table in front of the participants. The test was carried out under standardized conditions, e.g. defined luminance (160–320 cd/m^2^) and observation of the accurate seating position during the examination by trained and certified study nurses. The Nieden chart consists of seven different texts. The font size decreases and the word count increases between each text. Text no. 7 (visual acuity 0.32 at 40 cm) is written in the largest font size and has the smallest number of words whereas text no. 1 has the smallest font size (visual acuity 1.25 at 40 cm) with the largest number of words (word count from text no. 7 to text no. 1 respectively: 20, 26, 43, 42, 51, 61, 69). The time limit to read one text was 30 s. The test began with the text in the largest font size. If the participants could successfully read the first text, the next smaller text was uncovered.

(2) Secondly, reading comprehension was tested. One page of the local phone book was presented. The participants had to find the phone number of a defined general practitioner. The entry of name and telephone number was printed in font Arial Narrow in font size 7 pt.

Throughout both examinations the participants were asked to use viewing aids (e.g. reading glasses or lens) in case of refractive correction.

#### Quality of life

Quality of life was assessed via the German Version of the SF-12 questionnaire [25–29], a validated and reliable instrument to measure physical and mental factors of quality of life.

#### Daily activities, social participation, mobility, and falls

The questionnaires on daily activities, social participation and mobility were developed specifically for the SHIP-study. Seven aspects of daily activities were assessed in the survey: the participants were asked if they were able to: (1) go shopping independently, (2) handle their own financial affairs, (3) watch TV, (4) cross busy roads alone, (5) write a letter or postcard, (6) read a newspaper or magazine, (7) conduct simple crafting activities (e.g. sewing a button, hammering a nail). The participants could choose from three response options: ‘often’, ‘sometimes’ or ‘never’. If any of the responses to the seven items was ‘never‘, the observation was defined as a restriction of daily activities, all other response sets were rated as ‘not restricted’. Based on the responses, a score for everyday activities was calculated. Three points were assigned to ‘often’, two points to ‘sometimes’, and one point to ‘never’. The sum score of all seven questions ranged from seven to 21 points.

Eight items were used to assess social participation. The participants were asked whether they visited a sports club, a sports group, a hobby club or a health support group, had professional contacts, participated in church activities, participated in other groups or clubs (yes or no) or visited public events (often, sometimes or never). Not limited in social participation was defined if at least one all of eight items were answered with ‘yes’ or ‘often’ or ‘sometimes’. Observations with all eight items ‘no’ or ‘never’ were classified as ‘limited’.

Mobility was assessed by the participants’ use of different modes of locomotion (bike, moped, car, taxi, public transport, walking), as well as to the question whether they were usually accompanied or unaccompanied when they left their home. No restrictions in mobility were assumed if participants at least used one modus of locomotion and a person reported to go out unaccompanied. Mobility was considered restricted, if participants could only leave their home when accompanied, or when no means of locomotion were used (including no walking).

Falls were addressed by the question whether the participants had experienced at least one fall during the last 12 months.

#### Cognition

Assessment of cognition was performed with the Mini Mental State Examination (MMSE) [30]. The MMSE includes 23 items which aggregate to a maximum score of 30. MMSE is a widely used and valid instrument for measuring cognitive performance [31–34].

### Statistical analyses

The characteristics of the participants and the prevalences of cognitive reading ability (1) and reading comprehension (2) were analyzed with descriptive statistics. Bivariate correlations were calculated between quality of life, daily activities, mobility, social participation and cognitive reading ability (1), and reading comprehension (2), respectively, using Pearson product-moment, Spearman’s rank correlation coefficients, or a point biserial correlation dependent on the measurement scale.

Multivariable regression models were fitted to examine the relation of impaired cognitive reading ability (dependent variable) with quality of life, daily activities, social participation, and mobility (independent variables). Because of the distribution skewness of the values for cognitive reading ability, an additional sensitivity analysis with 3 categories (poor, moderate, and good cognitive reading ability) was performed. Another sensitivity analysis was carried out with other cut-off values for restrictions in daily activities.

All analyses were calculated with Stata Statistical Software, Release 14.1, StataCorp 2015.

## Results

### Descriptive statistics

We identified *N* = 780 participants of the SHIP-2 population who were 65 years of age or older (mean age 72.82 ± 5.91 years). Table 1 provides a summary of their basic characteristics. The gender ratio is near-balanced, the level of education was slightly lower in women.

**Table 1:** Basic characteristics of the participants

Characteristic	Male		Female		Total	
	n	(%)	n	(%)	n	(%)
Total	394	(100.00)	386	(100.00)	780	(100.00)
Age group						
65–69	128	(32.49)	140	(36.27)	268	(34.36)
70–74	116	(29.44)	124	(32.12)	240	(30.77)
75–79	85	(21.57)	74	(19.17)	159	(20.38)
80–84	46	(11.68)	34	(8.81)	80	(10.26)
85–89	18	(4.57)	13	(3.37)	31	(3.97)
> 89	1	(0.25)	1	(0.26)	2	(0.26)
Education (number of school years)						
< 10 years	225	(57.11)	246	(63.73)	471	(60.38)
10 years	87	(22.08)	88	(22.80)	175	(22.44)
> 10 years	79	(20.05)	52	(13.47)	131	(16.79)
Missing	3	(0.76)	0	(0.00)	3	(0.38)
MMSE score						
10–17	1	(0.25)	0	(0.00)	1	(0.13)
18–23	18	(4.57)	13	(3.37)	31	(3.97)
24–30	370	(93.91)	372	(96.37)	742	(95.13)
Missing	5	(1.27)	1	(0.26)	6	(0.77)
Limited mobility						
No	359	(91.12)	353	(91.45)	712	(91.28)
Yes	21	(5.33)	19	(4.92)	40	(5.13)
Missing	14	(3.55)	14	(3.63)	28	(3.59)
Falls within the last 12 months						
No	316	(80.20)	285	(73.83)	601	(77.05)
Yes	60	(15.23)	84	(21.76)	144	(18.46)
Missing	18	(4.57)	17	(4.41)	35	(4.49)
Restrictions in everyday activities						
No	282	(71.57)	320	(82.91)	602	(77.18)
Yes	98	(24.87)	52	(13.47)	150	(19.23)
Missing	14	(3.55)	14	(3.63)	28	(3.59)
Restrictions in social participation						
No	339	(86.04)	324	(83.94)	663	(85.00)
Yes	45	(11.42)	53	(13.73)	98	(12.56)
Missing	10	(2.54)	9	(2.33)	19	(2.44)

*MMSE* Mini Mental State Examination

Restrictions in social participation occurred more often in females. Women reported falls more often, whereas men experienced restrictions in daily activities more often. On average, the score of the MMSE in men (27.98 ± 2.18) and women (28.02 ± 2.00) was high and very similar between the sexes. In total, 91.1% of the participants had no limitation in mobility.

#### Functional reading ability

Functional reading ability was assessed on the basis of 2 examinations in 748 of 780 participants. Corrective lenses were used by 91.2% (*n* = 682) of the subjects during the examinations. Table 2 shows the results of both parts of the examination.

**Table 2:** Descriptive results of cognitive reading ability (Nieden) and reading comprehension by sex

Characteristic	Male		Female		Total		Mean in sec.	Median in sec.
	n	(%)	n	(%)	n	(%)		
Cognitive reading ability								
Text no 7 (largest font size)	375	(95.18)	366	(94.82)	741	(95.00)	8.14	7.59
Text no 6	350	(88.83)	347	(89.90)	697	(89.36)	15.51	14.63
Text no 5	271	(68.78)	280	(72.54)	551	(70.64)	21.94	21.74
Text no 4	197	(50.00)	204	(52.85)	401	(51.41)	22.11	21.96
Text no 3	34	(8.63)	26	(6.74)	60	(7.69)	26.47	27.31
Text no 2	4	(1.02)	1	(0.26)	5	(0.64)	26.81	26.57
Text no 1 (smallest font size)	3	(0.76)	0	(0.00)	3	(0.38)	26.50	26.51
No text could be read	3	(0.76)	4	(1.04)	7	(0.90)	–	–
Missing	16	(4.06)	16	(4.15)	32	(4.10)	–	–
Reading comprehension								
Ability to find a predefined phone number								
No	17	(4.31)	15	(3.89)	32	(4.10)		
Yes	361	(91.62)	355	(91.97)	716	(91.79)		
Missing	16	(4.06)	16	(4.15)	32	(4.10)		
Time to find a predefined phone number, in sec.								
≤ 10	58	(14.72)	68	(17.62)	126	(16.15)		
> 10 ≤ 20	110	(27.92)	123	(31.87)	233	(29.87)		
> 20 ≤ 30	71	(18.02)	67	(17.36)	138	(17.69)		
> 30 ≤ 40	42	(10.66)	34	(8.81)	76	(9.74)		
> 40 ≤ 50	29	(7.36)	20	(5.18)	49	(6.28)		
> 50	50	(12.69)	40	(10.36)	90	(11.54)		
Missing	34	(8.63)	34	(8.81)	68	(8.72)		

*sec* seconds

### Cognitive reading ability

Overall, 7.69% of the participants (*n* = 60) were able to read until text no. 3 within the maximum time of 30 s per text (good cognitive reading ability). Three participants were able to read the text with the smallest font size and the second smallest text was readable for two other participants (together 0.64%). 0.90% of the participants (*n* = 7) were unable to read any text.

Mean and median of the average time to read one text increased with decreasing font size (and thus also with increasing word count). The average time to read text no. 7 was 8.14 s (±2.63; median 7.59 s). Texts no. 3, 2 and 1 had an average reading time of approximately 26.50 (26.47, 26.81, 26.50) seconds.

### Reading comprehension

91.79% (*n* = 716) of the participants were able to find the predefined telephone number on the phone book page. The mean time to identify the phone number was 30.8 ± 35.25 s (median 20.00 s) with a range of 2 to 287 s. There were no differences by sex with respect to finding the telephone number but men needed more time on average.

**Fig. 1:**
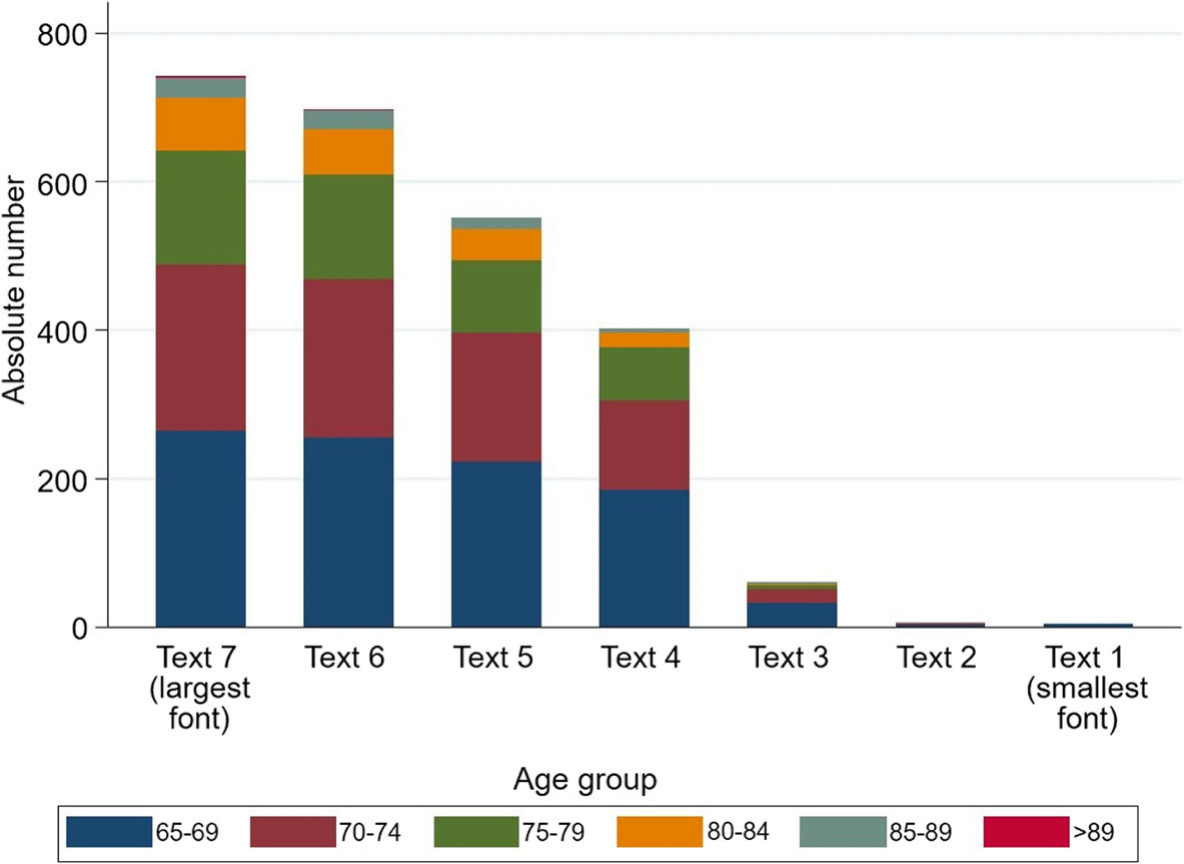
Cognitive reading ability measured on the basis of Nieden charts by age group

Table 3 and Fig. 1 show the results of functional reading ability by age. Increasing age was associated with a reduced maximum number of readable texts. However, this was not consistent with the results of the reading comprehension test. Participants who found the phone number were able to read texts in smaller font size on average.

**Table 3:** Descriptive results of cognitive reading ability bases of Nieden and reading comprehension by age

Characteristic	65–69		70–74		75–79		80–84		85–89		> 89	
	n	(%)	n	(%)	n	(%)	n	(%)	n	(%)	n	(%)
Cognitive reading ability												
Total	268	(100.00)	240	(100.00)	159	(100.00)	80	(100.00)	31	(100.00)	2	(100.00)
Text no 7 (largest font)	264	(98.51)	225	(93.75)	154	(96.86)	70	(87.50)	27	(87.10)	1	(50.00)
Text no 6	255	(95.15)	214	(89.17)	141	(88.68)	62	(77.50)	24	(77.42)	1	(50.00)
Text no 5	223	(83.21)	173	(72.08)	98	(61.64)	42	(52.50)	15	(48.39)	0	(0.00)
Text no 4	186	(69.40)	119	(49.58)	73	(45.91)	18	(22.50)	5	(16.13)	0	(0.00)
Text no 3	33	(12.31)	18	(7.50)	7	(4.40)	1	(1.25)	1	(3.23)	0	(0.00)
Text no 2	4	(1.49)	1	(0.42)	0	(0.00)	0	(0.00)	0	(0.00)	0	(0.00)
Text no 1 (smallest font)	3	(1.12)	0	(0.00)	0	(0.00)	0	(0.00)	0	(0.00)	0	(0.00)
no text could be read	1	(0.37)	4	(1.67)	0	(0.00)	1	(1.25)	1	(3.23)	0	(0.00)
Missing	3	(1.12)	11	(4.58)	5	(3.14)	9	(11.25)	3	(9.68)	1	(50.00)
Reading comprehension												
Able to find the predefined telephone number												
No	8	(2.99)	13	(5.42)	7	(4.40)	3	(3.75)	1	(3.23)	0	(0.00)
Yes	257	(95.90)	216	(90.00)	147	(92.45)	68	(85.00)	27	(87.10)	1	(50.00)
Missing	3	(1.12)	11	(4.58)	5	(3.14)	9	(11.25)	3	(9.68)	1	(50.00)

### Bivariate correlations

A positive bivariate correlation was found between reading comprehension and cognitive reading ability (coefficient: 0.35, *p* < 0.001). No significant associations were found between reading comprehension and quality of life, daily activities, mobility, social participation, and education.

The ability of reading text in smaller fonts was associated with a higher level of education (R_S_ = 0.24, *p* < 0.001), a higher score in MMSE (R_S_ = 0.22, p < 0.001) and fewer restrictions in daily activities (R_S(bis)_ = 0.22, *p* < 0.001). Numerically weak but statistically significant correlations were found between cognitive reading ability and the physical component of quality of life (R_S_ = 0.16, *p* < 0.001) and between cognitive reading ability and mobility (R_S(bis)_ = 0.14, p < 0.001).

### Multivariate regression models

#### Quality of life

Table 4 shows the results of the multivariate regression models for quality of life. Having a better cognitive reading ability was not a significant determinant for a better physical component of quality of life. Increasing age, falls in the previous 12 months, and limited mobility were negative determinants for the physical component of quality of life. A higher score for daily activities improved the physical component of quality of life.

**Table 4:** Results of the linear regression analyses, outcome quality of life

	PCS, SF12		MCS, SF12	
	Regression coefficient	*P*-Value	Regression coefficient	P-Value
Text number 7 (largest font size)	0.20	0.958	0.06	0.985
(reference: no text could be read)	[−7.15,7.54]		[−6.49,6.61]	
Text number 6	−2.75	0.437	−1.80	0.569
	[−9.69,4.19]		[− 7.99,4.39]	
Text number 5	−2.91	0.411	−1.24	0.694
	[−9.86,4.04]		[−7.44,4.96]	
Text number 4	− 1.81	0.605	− 1.16	0.711
	[−8.68,5.06]		[−7.29,4.97]	
Text number 3	−1.40	0.707	0.54	0.872
	[−8.70,5.90]		[−5.98,7.05]	
Text number 2	4.31	0.551	5.50	0.394
	[−9.88,18.50]		[−7.16,18.16]	
Text number 1 (smallest font size)	−12.92	0.075	2.39	0.712
	[−27.14,1.30]		[−10.30,15.07]	
Age	−0.27	< 0.001	0.03	0.589
	[−0.40,-0.14]		[−0.08,0.15]	
Sex	−1.15	0.103	−2.79	< 0.001
(reference: men)	[−2.54,0.23]		[−4.03,-1.56]	
Education: 10 school years	1.05	0.227	1.80	0.021
(reference: < 10 school years)	[−0.66,2.76]		[0.28,3.32]	
> 10 school years	0.14	0.886	−1.01	0.253
	[−1.80,2.09]		[−2.75,0.72]	
MMSE score	0.32	0.101	0.05	0.790
	[−0.06,0.71]		[−0.30,0.39]	
Falls	−3.77	< 0.001	−2.19	0.007
(reference: no falls)	[−5.54,-1.99]		[−3.77,-0.60]	
Limited mobility	−4.10	0.020	−1.18	0.449
(reference: not restricted)	[−7.54,-0.66]		[−4.25,1.89]	
Score of everyday activities	1.22	< 0.001	1.03	< 0.001
	[0.75,1.68]		[0.62,1.44]	
Limited in social participation	−0.25	0.818	−1.48	0.132
(reference: not restricted)	[−2.42,1.91]		[−3.42,0.45]	
Constant	34.01	< 0.001	32.82	< 0.001
	[16.77,51.24]		[17.44,48.19]	
*R* ^2^	0.184		0.123	
Adjusted *R*^2^	0.164		0.101	
Observations	663		663	

95% confidence intervals in brackets

*SF* Short Form, *PCS* Physical Component Summary, *MCS* Mental Component Summary, *MMSE* Mini Mental State Examination

The multivariable model for the mental component of quality of life also showed no significant influence of cognitive reading ability. A greater ability to perform daily activities increased mental quality of life. Being female was associated with a lower mental quality of life and with having falls.

#### Daily activities, social participation and mobility

To investigate whether a better cognitive reading ability is a determinant for having no restrictions in daily activities, social participation, and mobility, three multivariable logistic regression models were calculated. The results are shown in Table 5.

**Table 5:** Results of the logistic regression models

	Daily activities		Social participation		Mobility	
	Odds ratio	*P*-Value	Odds ratio	*P*-Value	Odds ratio	*P*-Value
Text number 7 (largest font size)	2.94	0.273	0.46	0.530	2.01	0.540
(reference: no text could be read)	[0.43,20.28]		[0.04,5.24]		[0.22,18.76]	
Text number 6	4.55	0.108	0.44	0.489	2.05	0.501
	[0.72,28.83]		[0.04,4.59]		[0.25, 16.69]	
Text number 5	6.71	0.044	0.41	0.462	3.45	0.262
	[1.05,42.90]		[0.04,4.40]		[0.40, 30.08]	
Text number 4	8.53	0.025	0.63	0.700	3.54	0.242
	[1.36,53.37]		[0.06,6.69]		[0.43, 29.41]	
Text number 3	5.82	0.080	0.26	0.280	2.88	0.410
	[0.81,41.76]		[0.02,3.04]		[0.23, 35.82]	
Text number 2	empty		empty		empty	
Text number 1 (smallest font size)	empty		empty		empty	
Age	0 .98	0.188	0.97	0.087	0.96	0.144
	[0.94, 1.01]		[0.93,1.01]		[0.90, 1.01]	
Sex	2.13	< 0.001	0.70	0.152	0.77	0.491
(reference: men)	[1.40, 3.25]		[0.43,1.14]		[0.37, 1.61]	
10 school years	1.39	0.225	1.49	0.152	0.79	0.622
(reference: < 10 school years)	[0.82, 2.39]		[0.80,2.78]		[0.31, 2.00]	
> 10 school years	2.40	0.014	2.84	0.024	0. 71	0.537
(reference: < 10 school years)	[1.19, 4.85]		[1.15,7.02]		[0.23, 2.13]	
MMSE score	1.22	< 0.001	1.05	0.436	1.12	0.176
	[1.11, 1.35]		[0.93,1.18]		[0.95, 1.31]	
Daily activities	–	–	3.27	< 0.001	6.94	< 0.001
(reference: restricted)	–		[1.90,5.61]		[3.17, 15.19]	
Limited mobility	–	–	0.63	0.283	–	
(reference: not restricted)	–		[0.27,1.47]		–	
Falls	0.43	0.001	1.13	0.693	0.55	0.134
(reference: no falls)	[0.27,0.84]		[0.62,2.07]		[0.25, 1.20]	
Pseudo *R*^2^	0.12		0.10		0.19	
Observations	707		704		705	

95% confidence intervals in brackets

*MMSE* Mini Mental State Examination

The chance not to be restricted in daily activities increases with a better cognitive reading ability. Being female, have a better MMSE score and no falls during the last 12 months were determinants for no restrictions in daily activities.

There was no significant influence of cognitive reading ability on social participation. The chance of being not limited in social participation was better with higher education (> 10 school years) and with no restrictions in daily activities.

Not being restricted in daily activity is a significant determinant for mobility (OR 6.94, *p* < 0.001).

#### Sensitivity analysis

To take the skewness of the values of the cognitive reading ability into account, participants were assigned to three categories in an additional sensitivity analysis. The first group included subjects with severely impaired cognitive reading ability (not able to read any text or able to read texts no. 7 or no. 6, *n* = 197) only. The second group included subjects with cognitive reading ability for texts no. 5 and 4 (*n* = 491, moderate cognitive reading ability). The third group (*n* = 60) consisted of individuals who were able to read the texts no. 3, 2 or 1 (good cognitive reading ability). The ability to perform daily activities was significantly associated with moderate cognitive reading ability (OR 1.73, *p* = 0.023).

Another sensitivity analysis was carried out with another cut-off value for restrictions in daily activities. Participants were defined as restricted in daily activities if they never did at least two of the given activities. However, since only *n* = 19 participants (2.44%) were restricted according to this cut-off value, no further statistical analysis was conducted with daily activities as dependent variable. If the additional classification is included into the regression models as an independent variable, restrictions in daily activities are a determinant of restrictions in mobility (OR 10.57; 95% confidence interval: 2.67, 41.86; *p* = 0.001).

## Discussion

To investigate cognitive reading ability, Nieden’s near vision reading charts with two measurement dimensions were used. It has previously been shown that standard single-optotype acuity charts offer only limited potential to evaluate reading ability [35, 36]. The advantage of reading charts is their ability to provide valid measurements of reading ability parameters [37]. In comparison to a single-optotype acuity chart, reading charts resemble the reading behaviour in daily life more closely. A healthy eye should be able to read Nieden 1 (text with the smallest font size and the highest number of words). In case of tiredness or distraction, a maximum of reading texts 2 and 3 is still acceptable. In this study only 7.69% (*n* = 60) of the participants were able to read texts 1, 2 or 3.

The results of the sensitivity analysis (using three categories of cognitive reading ability) show a positive effect of higher reading ability on performing daily activities.

5.0% (*n* = 37) of the participants had corrective lenses in daily life but did not use them in the functional reading ability examination. It can be assumed that subjects could have read smaller texts if they had used their glasses. An improvement would also have been likely if more than 30 s time had been allowed.

Loss of reading ability is a slow and progressive, age-related process and individuals may not be aware of changes in their sight. In addition, some degree of loss may be compensated for and thus may not impact the quality of life, daily activities, social participation and mobility.

The ability to perform daily activities is a key indicator for an autonomous lifestyle. The ability to perform daily activities is influenced by cognitive ability, social participation, mobility and falls during the last 12 months. In the multivariable models, age was not associated with the performance of daily activities in this study; this may at least partly be explained by a cohort effect: this means that the study population is on average healthier than the general population. In addition, the MMSE measure appears to be less suitable for detecting mild cognitive impairment [38] because of its ceiling and bottom effects [32].

Previous studies have investigated (near) visual acuity in relation to various outcome factors. The results of a cross-sectional study of visually impaired participants ≥55 years showed no association for severity, duration and primary cause of visual impairment with social participation [39]. However, in a study among community-dwelling elderly aged ≥65 years, Desrosiers et al. found that visual impairment was related to lower levels of participation of those affected compared to subjects without visual impairment. However, visual acuity did not correlate with social participation [8]. Similarly, Crews et al. showed that older people with visual impairment, defined as blindness in one or both eyes or having trouble to see even with glasses, had a lower level of participation and they wished to have a higher level of social activity compared with those who were not visually impaired [7]. In further studies, an association between visual impairment and social participation could not be shown [39].

Aartolahti et al. assessed functional vision based on a functional vision questionnaire. They also assessed cognition by the MMSE and found a lower cognition in participants with low functional vision compared to those with moderate or good functional vision [16]. This is in accordance with the positive association of MMSE score and reading ability in our analysis. Cognition is important for mobility performance, whereas visual acuity, contrast sensitivity or dynamic vision were not associated with the mobility performance, as identified by Patel et al. [40].

Functional reading ability in the general elderly population has been rarely examined so far. In the Salisbury Eye Evaluation (SEE) reading speed and having difficulties to read a newspaper were evaluated. Reading speed in this study ranged from 169 to 174 words per minute [41]. Text no. 1 in this study (text in smallest font size and with most words) consisted of 69 words (time limit: 30 s). Other studies use standardized reading speed tests [35, 42, 43]. However, the rate of word reading is not fully comparable with the ability to recognize words and understand the meaning of the text.

Selection effects might have biased the results. The participants of this cohort study had to visit the examination center in person and had to conduct a comprehensive examination program. People with limitations in mobility or cognition or illiterate persons were presumably less likely to travel to participate.

## Conclusion

The prevalence of reduced functional reading ability was high in the elder age groups in the general population. However, the impact on daily activities, social participation, mobility, and quality of life was limited in the study population. Further research is needed and planned, including populations with a wider range of comorbidity, and different degrees of frailty.

## Data Availability

The usage of SHIP data must be applied at the transfer office for data and biomaterial management at the University Medicine Greifswald (https://www.fvcm.med.uni-greifswald.de/dd_service/data_use_intro.php?lang=ger).

## Abbreviations

ICF: International Classification of Functioning, Disability and Health
MCS: Mental Component Summary
MMSE: Mini Mental State Examination
PCS: Physical Component Summary
